# Supplementary material for: Phononic bath engineering of a superconducting qubit
Source: Nat Commun. 2023 Jul 3;14:3910. doi: 10.1038/s41467-023-39682-0 (PMC10318091; doi:10.1038/s41467-023-39682-0)
Supplement: Supplementary file 1 — Supplementary Info [file 41467_2023_39682_MOESM1_ESM.pdf]

SUPPLEMENTARY INFORMATION

SUPPLEMENTARY NOTE 1: SAW RESONATOR DESIGN AND MODELING

The spectral response of the SAW resonator was calculated using coupling-of-modes (COM) [1]. The periodicity of the SAW interdigitated transducer (IDT) structure is fabricated to be $\lambda_{\mathrm{IDT}} = 800$ nm. The periodicity of the mirror structure is fabricated to be slightly larger than the periodicity of the IDT structure ($\lambda_{\mathrm{mirror}} = 816$ nm) to guarantee a single sharply peaked resonant acoustic mode within the stopband of the mirrors where phonons are strongly reflected, which produces the confined acoustic resonance at 4.46 GHz. A list of the complete SAW device parameters are listed in Supplementary Table 1. The last four rows of Supplementary Table 1 are adjusted to fit the spectroscopy of the hybrid system (see Fig. 2(a,b) in the main text) to the simulated conductance via coupling-of-modes. The first six rows of Supplementary Table 1 define the geometric properties of the SAW device and are defined in fabrication, and we calculate the relevant scattering parameters for each structure in the SAW resonator using coupling-of-modes with the results shown in Supplementary Fig. 1.

Parameter	Physical quantity	Value
λ_{IDT}	Transducer periodicity	800 nm
$\lambda_{\mathrm{mirror}}$	Mirror periodicity	816 nm
N_P	Number of finger pairs	16
W	Finger pair overlap	35 μm
L_{mirror}	Bragg mirror length	240.72 μm
L_{IDT}	Transducer length	12 μm
v_{sound}	Speed of sound	3638 m/s
η	SAW propagation loss	500 Np/m
r_i	Transducer reflectivity	-0.005i
r_m	Mirror reflectivity	-0.005i

Supplementary Table I: **Acoustic resonator parameters.** Summary of relevant parameters for the SAW resonator used in the experiment

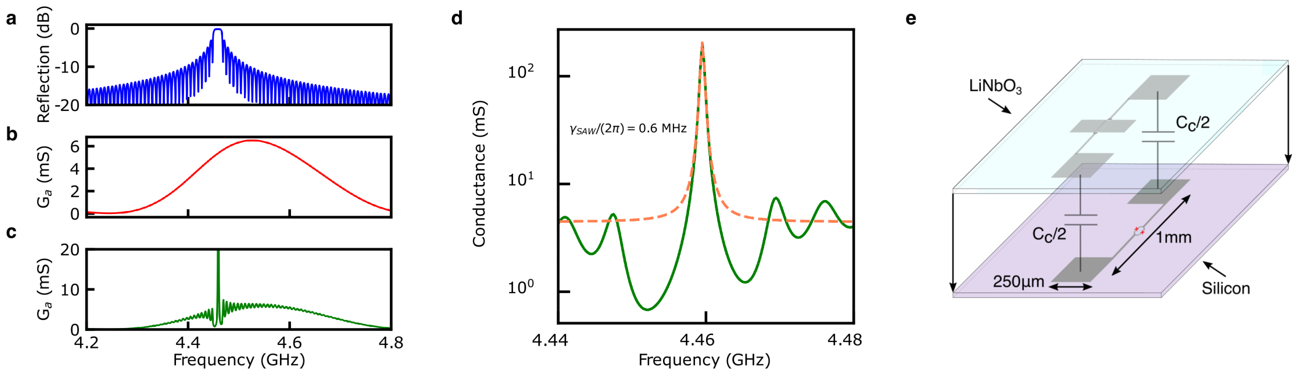

Supplementary Figure 1: **Modeling of the SAW device and its coupling to the qubit.**(a) Simulated reflection of the Bragg mirror structure in the SAW resonator. (b) Simulated conductance of the SAW transducer, which is centered between the two acoustic Bragg mirrors. (c) Composite conductance of the SAW transducer cascaded with the mirror structure, leading to a single confined SAW mode near 4.46 GHz and a continuous conductance associated with the leakage of SAW energy through the Bragg mirrors. (d) Near the confined mode the SAW resonator conductance can be approximated by a Lorentizian function (dashed orange fit). The full width at half maximum of this Lorentizian is used to estimate the energy decay rate of the confined SAW mode. (e) Schematic of the flip-chip assembly. The antenna pads on either substrate form parallel plate capacitors between the two devices.

The broad frequency response of the conductance in Supplementary Fig. 1(c) arises from the Fourier transform of the spatial structure of the SAW transducer. The width of the response is dictated by the number of finger pairs constituting the transducer, labeled N_p in Supplementary Table 1 (see Fig. 1 in the main text) [2]. The SAW device is galvanically connected to two large antenna pads and coupled to the transmon qubit via a flip-chip technique, forming an effective set of parallel plate capacitors between the antenna pads on the SAW and transmon chips as depicted in Supplementary Fig. 1(e).

SUPPLEMENTARY NOTE 2: NUMERICAL CALCULATION OF LINDBLAD DYNAMICS

Given a periodic drive coupling to the σ_x operator with Rabi frequency Ω, the Hamiltonian that describes the qubit in the frame rotating at ω_d is given by

$$H = \frac{\Delta}{2}\sigma_z + \frac{\Omega}{2}\sigma_x, \tag{1}$$

where $\Delta = \omega_d - \omega_q$ is the qubit-drive detuning. In the presence of the drive, the corresponding qubit eigenstates ($|g\rangle$,$|e\rangle$) are rotated to a dressed basis ($|\tilde{g}\rangle$,$|\tilde{e}\rangle$), corresponding to the eigenvectors of Supplementary Eq.1:

$$|\tilde{g}\rangle = \cos\theta\,|g\rangle - \sin\theta\,|e\rangle \tag{2}$$
$$|\tilde{e}\rangle = \sin\theta\,|g\rangle + \cos\theta\,|e\rangle\,,$$

where the rotation angle of the eigenstates is defined by $\tan 2\theta = -\Omega/\Delta$. This allows the Hamiltonian to be rewritten in the dressed basis as

$$H = \frac{\Omega_R}{2}\tilde{\sigma}_z, \tag{3}$$

where $\Omega_R = \sqrt{\Omega^2 + \Delta^2}$ is the generalized Rabi frequency and $\tilde{\sigma}_z = \sin 2\theta\sigma_x - \cos 2\theta\sigma_z$. The evolution of the reduced density matrix in the dressed basis as given by the Lindblad master equation [3]

$$\dot{\rho} = i[\rho, H] + \gamma_0 \cos^2(\theta)\sin^2(\theta)\mathcal{D}[\tilde{\sigma}_z]\rho + \gamma_- \sin^4(\theta)\mathcal{D}[\tilde{\sigma}_+]\rho +$$
$$\gamma_+ \cos^4(\theta)\mathcal{D}[\tilde{\sigma}_-]\rho + \gamma_1\mathcal{D}[\sigma_-]\rho + \frac{\gamma_\phi}{2}\mathcal{D}[\sigma_z]\rho. \tag{4}$$

In Supplementary Eq. 4 ρ is the reduced density matrix, which describes the driven two level system, and $\mathcal{D}[A]\rho = \left(2A\rho A^\dagger - A^\dagger A\rho - \rho A^\dagger A\right)/2$. The rates γ_+ and γ_- are the transition rates between qubit eigenstates in the dressed basis, which sample the qubit loss at the frequencies $\omega_d \pm \Omega_R$, and γ_0 is the dephasing rate in this basis. Qubit dynamics are also impacted by global depolarization and dephasing rates γ_1 and γ_ϕ, respectively. We represent the density matrix as a vector $\rho = (\rho_{gg}, \rho_{ge}, \rho_{eg}, \rho_{ee})^T$, which is evolved in time as $\rho(t) = e^{\mathcal{L}t}\rho(0)$. The matrix $\mathcal{L}$ represents the Lindblad operator as a 4×4 matrix that is exponentiated via the transformation $Ve^{Dt}V^{-1}$, where V is the matrix that diagonalizes $\mathcal{L}$ and D is a diagonal matrix containing the eigenvalues of $\mathcal{L}$. We calculate expectation values by taking the trace $\langle\sigma_x\rangle = \mathrm{Tr}(\sigma_x\rho)$ and similarly for the other components of the qubit expectation value.

SUPPLEMENTARY NOTE 3: CALIBRATIONS

We calibrate the magnitude of the resonant Rabi frequency by measuring the excited state population of the qubit as a function of time over a range of drive amplitudes as shown in

Supplementary Fig. 2(a). The frequency of the resulting oscillatory behavior varies linearly with drive amplitude as shown in Supplementary Fig. 2(b) and provides a mapping between Rabi frequency and drive. A more detailed description of the experimental measurement setup can be found in [4].

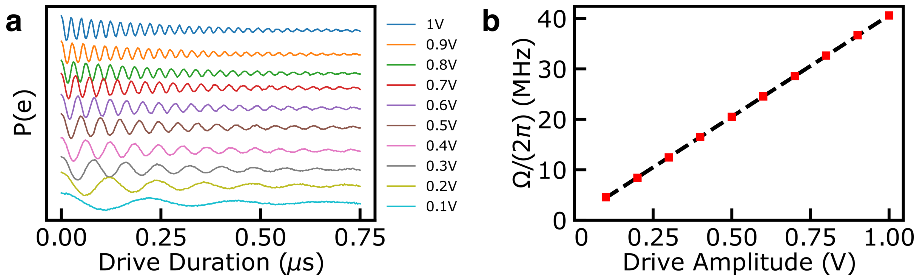

Supplementary Figure 2: **Calibration of resonant Rabi oscillations.** (a) Resonant Rabi oscillations for various drive amplitudes ranging from 0.1V to 1V. As the amplitude is increased, the frequency of oscillation increases. Traces have been vertically offset for clarity. (b) Extracted Rabi frequencies from the data in panel (a). The frequency increases linearly with drive amplitude as expected and allows us to interpolate the resonant Rabi frequency for a given drive amplitude.

To determine the pure dephasing rate relevant to the measurements presented in Figures 3-5 of the main text, we measure the qubit lifetime and Ramsey decay at a qubit frequency of $\omega_q/(2\pi) = 4.001$ GHz as shown in Supplementary Fig. 3. From these measured values we calculate the pure dephasing rate as

$$\gamma_{\phi,\mathrm{exp}} = \frac{1}{T_2^*} - \frac{1}{2T_1}, \tag{5}$$

and find $\gamma_{\phi,\mathrm{exp}} = 0.93\ \mu\mathrm{s}^{-1}$, in reasonable agreement with the fitted value of $1.48\ \mu\mathrm{s}^{-1}$.

SUPPLEMENTARY NOTE 4: TOMOGRAPHY DATA AND ANALYSIS

To determine the steady-state value of $\langle\sigma_x\rangle$ and the purity of superposition states of the qubit shown in Figure 4 and 5 of the main text, a coherent drive is applied to the system for $t_{\mathrm{drive}} = 3\ \mu$s. The qubit state is then reconstructed via full state tomography. For completeness we also present the measured values of $\langle\sigma_z\rangle$ and $\langle\sigma_y\rangle$ in Supplementary Fig. 4(a,c) along with comparison to Supplementary Eq. 4 in Supplementary Fig. 4(b,d). The qubit reduced density matrix is given by $\rho = \frac{1}{2}\left(\mathbb{1} + \sigma_x\langle\sigma_x\rangle + \sigma_y\langle\sigma_y\rangle + \sigma_z\langle\sigma_z\rangle\right)$, where $\mathbb{1}$

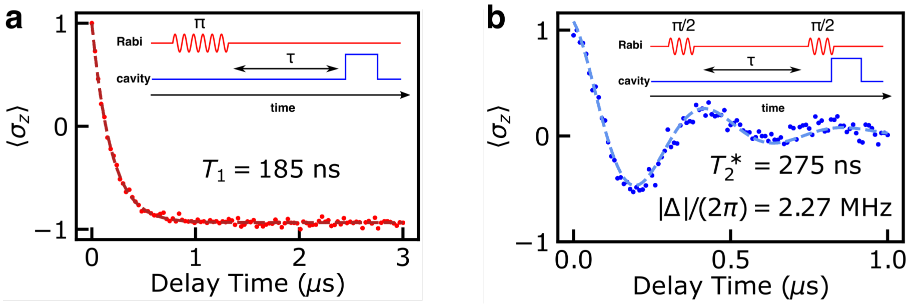

Supplementary Figure 3: **Qubit depolarization and dephasing times.**(a) Measurement of qubit lifetime T_1 at $\omega_q/(2\pi) = 4.001$ GHz. (b) Measurement of the Ramsey decay T_2^* at the same qubit frequency with a drive detuned by $|\Delta|/(2\pi) = 2.27$ MHz from the qubit.

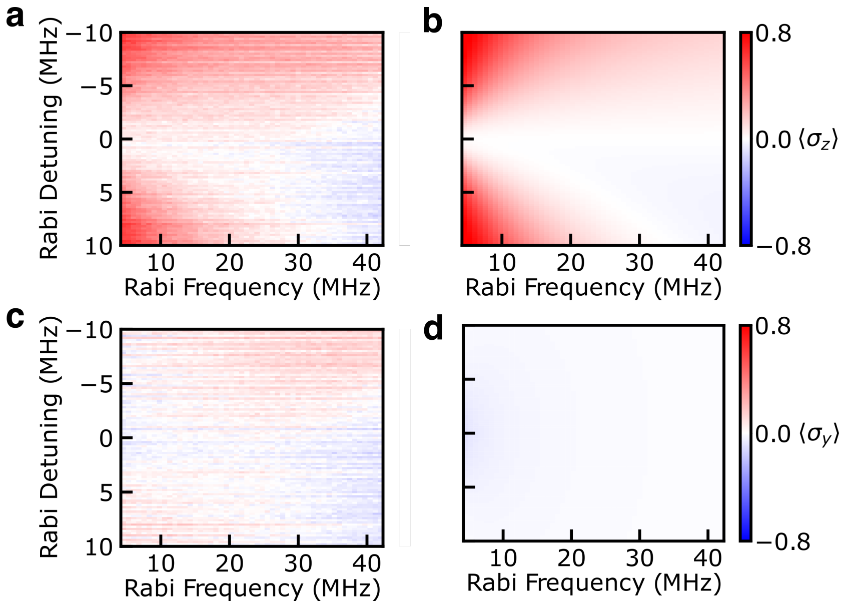

Supplementary Figure 4: **Remaining qubit state tomography components**(a) Measured values of $\langle\sigma_z\rangle$ with the same drive parameters as in Fig. 4 of the main text. (b) $\langle\sigma_z\rangle$ obtained from solutions to Supplementary Eq. 4 with the same drive parameters as Fig. 4 of the main text. (c) Measured values of $\langle\sigma_y\rangle$ with the same drive parameters. (d) $\langle\sigma_y\rangle$ obtained from solutions to Supplementary Eq. 4 with the same drive parameters.

is the 2×2 identity matrix. Each tomography component is calibrated relative to the readout contrast along each axis of the Bloch sphere and scaled such that the length of the qubit state vector does not exceed unity. The purity of a given state is then calculated to be $\mathcal{P} = \mathrm{Tr}(\rho^2)$ and is shown in Fig. 5(a,c) of the main text and is compared with the results of Supplementary Eq.4 in Fig. 5(b,c). The systematic difference between the measured

tomography components and those obtained from solutions to Supplementary Eq. 4 (most evident as small non-zero measured values of $\langle\sigma_y\rangle$ in Supplementary Fig. 4(c)) likely arises from systematic errors produced by time-dependent variation of the qubit transition frequency over the relatively long duration (several hours) of the tomography measurements as a function of drive parameters. A tomography measurement consists of a $\pi/2$ rotation with a well-defined phase to project either the x or y component of the qubit state vector onto the measurement (z) axis of the Bloch sphere, and subsequent projective measurement. To obtain the z-component of the state vector, no tomography rotation is required prior to measurement. In the presence of a detuned drive, as is the case for our measurements at $\Delta \neq 0$ in Figs. 3-5 in the main text and Supplementary Fig. 4, the qubit state acquires an additional phase shift of $\phi = 2\pi \times \Delta \times t_{\text{drive}}$, during the detuned drive. To account for this additional phase accumulation, the phase of each tomography pulse is shifted by the same amount ϕ prior to projective measurement. Because the phase of the tomography pulses depend on the detuning in this fashion, fluctuations in Δ introduce systematic errors in the measured tomography components. Qubit frequency variations can be clearly seen in measurements of Ramsey interferometry taken over many hours, as shown in Supplementary Fig. 5. We observe slow drift in the qubit frequency, indicating the presence of low-frequency noise in the system as well as discrete "jumps" in the qubit frequency. These type of discrete

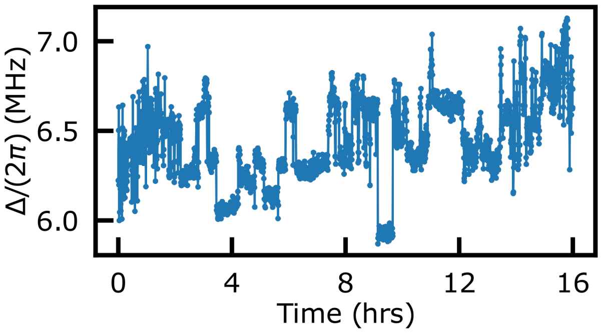

Supplementary Figure 5: **Representative measurement of qubit frequency fluctuations.** Detuning ($\Delta = \omega_d - \omega_q$) between the time-varying qubit frequency and a fixed drive frequency ($\omega_d/(2\pi) = 4.007$ GHz) measured via Ramsey interferometry over 16 hours. Both slowly varying frequency drift and discrete frequency jumps on the order of 1 MHz are observed in $\omega_q/(2\pi)$. The discrete frequency jumps are likely due to non-resonant coupling to two-level fluctuators in the qubit chip.

jumps are often seen in superconducting qubit experiments and in our case likely arise from unwanted coupling to dielectric two-level fluctuators [5].

SUPPLEMENTARY REFERENCES

[1] J. R. Lane, *Integrating Superconducting Qubits with Quantum Fluids and Surface Acoustic Wave Devices*, Ph.D. thesis, Michigan State University (2021).

[2] D. P. Morgan, *Surface Acoustic Wave Devices*, 2nd ed. (Elsevier Ltd., 2005) https://onlinelibrary.wiley.com/doi/pdf/10.1002/0471654507.eme434.

[3] G. Lindblad, On the generators of quantum dynamical semigroups, Communications in Mathematical Physics **48**, 119 (1976).

[4] J. R. Lane, D. Tan, N. R. Beysengulov, K. Nasyedkin, E. Brook, L. Zhang, T. Stefanski, H. Byeon, K. W. Murch, and J. Pollanen, Integrating superfluids with superconducting qubit systems, Phys. Rev. A **101**, 012336 (2020).

[5] C. Müller, J. H. Cole, and J. Lisenfeld, Towards understanding two-level-systems in amorphous solids: insights from quantum circuits, Reports on Progress in Physics **82**, 124501 (2019).
